# Viewpoints about collaboration between primary care and public health in Canada

**DOI:** 10.1186/1472-6963-13-311

**Published:** 2013-08-14

**Authors:** Noori Akhtar-Danesh, Ruta Valaitis, Linda O’Mara, Patricia Austin, Val Munroe

**Affiliations:** 1 School of Nursing, McMaster University, Hamilton, Ontario, Canada; 2 Department of Clinical Epidemiology & Biostatistics, McMaster University, Hamilton, Ontario, Canada; 3 Vancouver Coastal Health, Vancouver, British Columbia, Canada

**Keywords:** Public health, Primary care, Collaboration, Partnership, Primary health care, Policy makers, Q-methodology

## Abstract

**Background:**

Although there is a global movement toward health system integration and collaboration, little is known about values, beliefs, and attitudes towards collaboration between stakeholders in public health (i.e. promotion, protection, and prevention with vulnerable groups and/or at the population level) and primary care (i.e., family practices, nurse-led clinics). The purpose of this study was to explore viewpoints of key stakeholders regarding primary care (PC) and public health (PH) collaboration in Canada.

**Methods:**

We used Q-methodology to identify common viewpoints held by participants who attended a national meeting in Canada in 2010 to discuss PC and PH collaboration. The study was conducted in two phases. In Phase 1 a Q-sample, a Q-sort table, and a short demographic questionnaire were developed which were used in Phase 2 for data collection. The Q-sorts then were analysed to identify the salient factors and consensus statements.

**Results:**

In total, 25 multidisciplinary individuals including researchers, policy-makers, directors, managers, and practitioners (e.g., nurses, family physicians, dietitians) participated. Using a by-person factor analysis, three factors (salient viewpoints) emerged. Factors were named based on their distinguishing statements as follows: a) System Driven Collaborators, b) Cautious Collaborators, and c) Competent Isolationists. System Driven Collaborators strongly believed that a clear mandate from the top is needed to enable PH, PC and the rest of the health system to effectively work together and that people in different branches in the Ministry/ Ministries have to strongly believe in collaboration, actively support it, and develop directed policies to foster organizations work together. Cautious Collaborators strongly supported the idea of having better consciousness-raising about what collaborations might be possible and beneficial, and also reflecting on the collaborations already in place. The Competent Isolationists strongly believed that it is necessary for PC and PH sectors to spend time to ensure that both parties clearly understand the differences between their roles. They believe that physicians, nurses, and social workers will not see the value in collaboration because they lack inter-professional educational programs.

**Conclusions:**

Different viewpoints are held by stakeholders around PC and PH collaboration which have the potential to influence the success of collaborations. Understanding and managing these differences is important to assist change management processes required to build and maintain strong PC and PH collaborations.

## Background

Improved collaboration between primary care (PC) and public health (PH) sectors can lead to a stronger understanding of the communities that they serve and lead to more responsive and comprehensive delivery of health services [1]. Further, “integration of PC and PH could enhance the capacity of both sectors to carry out their respective missions and link with other stakeholders to catalyze a collaborative, intersectoral movement toward improved population health” (p.1) [2]. The World Health Organization directs nations to foster “reforms that secure healthier communities, by integrating public health actions with primary care and by pursuing healthy public policies across sectors” [3]. Worldwide, there is a growing body of literature about factors influencing the building and maintenance of collaborative relationships between PH (i.e. promotion, protection, and prevention with vulnerable groups and/or at the population level) and PC (i.e., family practices, nurse-led clinics) [1,2]. However, there is still very little known about stakeholders’ views that have the potential to positively or negatively influence these relationships. The purpose of this study was to explore viewpoints of key stakeholders regarding PC and PH collaboration in Canada.

### The concept of collaboration

This study is part of a larger program of research on PC and PH collaboration which involved 5 studies including: 1) a scoping literature review [1] which examined international literature; 2) key informant interviews with over 70 stakeholders from across Canada; 3) three provincial environmental scans; 4) ten in-depth case studies of PC and PH collaboration in three provinces; and, 5) the Q-sort study reported here. The program of research focused on exploring factors influencing PC and PH collaboration in Canada, the results of which informed the development of an ecological framework for successful PC and PH collaboration [4]. This framework built upon San-Martin Rodriques and colleagues [5] framework which explored systemic, organizational, and interactional factors influencing interorganizational collaboration. The large multijurisdictional multi-disciplinary research team which included academic researchers, PC and PH providers, managers and provincial policy makers defined collaboration. For the purposes of this program of research, the team defined collaboration as per the Public Health Agency of Canada’s (PHAC) definition as - “a recognized relationship among different sectors or groups, which have been formed to take action on an issue in a way that is more effective or sustainable than might be achieved by the public health sector acting alone” (p. 9) [6]. The term *collaboration* was conceptualized as a point on a continuum of ways of working together that includes: networking, cooperation, coordination, and collaboration. These levels of working together are differentiated by Himmelman [7], and are based on a mix of elements including: exchanging information, altering activities for mutual purpose, sharing resources, and enhancing the capacity of another for mutual benefit and to achieve a common purpose.

PC can be considered one of primary health care’s core services. We used Barbara Starfield’s definition of PC as, “that level of a health service system that provides entry into the system for all new needs and problems, provides person-focused (not disease-focused) care over time, provides care for all but very uncommon or unusual conditions, and coordinates or integrates care provided elsewhere or by others” (p. 8–9) [8].

Similar to PC, PH can also be considered one of primary health care’s core services. As per the Public Health Agency of Canada, we defined PH as: “…an organized activity of society to promote, protect and improve, and when necessary, restore the health of individuals, specified groups, or the entire population. It is a combination of sciences, skills, and values that function through collective societal activities and involve programs, services, and institutions aimed at protecting and improving the health of all people.” (p.13) [6].

#### Why PH and PC collaboration?

The Institute of Medicine’s Committee on Integrating Primary Care and Public Health states that collaboration can increase efficiencies and effectiveness of PC and PH functions, and lead to collaboration with others towards improvement of population health [2]. In the U.S., Lasker and the Committee on Medicine and Public Health [9] identified numerous ways that PC and PH collaboration can improve health care systems-- by improving coordination of care, increasing access to care for the uninsured, leveraging clinical practice to help identify and address community health problems, strengthening promotion and prevention campaigns, improving cost-effectiveness and quality of PC through the application of population health approaches, and finally, increasing collaboration efforts pertaining to policy training and research. Lebrun and colleagues examined health centres in the U.S. that provide a safety net for PC and PH services for the uninsured [10]. They found cases of collaborative activities ranging from monitoring population health status, mobilizing community partnerships around health problems, educating and advocating for laws protecting health, to developing policies to support individual and community health efforts. Green and colleagues argue that it is imperative for PC and PH as well as others to integrate their services to improve prevention efforts for diseases such as diabetes [11]. In Brazil, PC and PH collaborations have been shown to enhance support provided to underserved populations, and encourage community-focused, trans-disciplinary approaches in the delivery of programs and services [12]. In Canada, in-depth case studies of collaboration have been explored that resulted in enhanced professional capacity building related to tobacco cessation and 18 month enhanced well baby visits; more effective regional vaccine and immunization management; community betterment through collaborative health promotion programming; and improved access to care through outreach to vulnerable populations [4]. In Alberta, Oelke argued that, “the enhanced collaboration between all components of the primary care system has increased the efficiency of service delivery and provided an opportunity to involve services that were previously absent” (p.78) [13].

Despite evidence that PC and PH collaborations have resulted in positive health care system outcomes, health professional outcomes, and health benefits to individuals and populations [1] collaboration is not the norm seen in practice. Furthermore, policy makers are calling for more strategic action towards stronger integration of health systems [14,15]. It has been suggested that there is much potential for alignment of these two sectors as their aims are complementary [16], although their approaches, education and perspectives differ [17]. Thus it is not surprising that barriers and facilitators have been identified related to building and maintaining PC and PH collaboration.

An ecological perspective applied in this program of research and the resulting framework that was developed illustrated factors at the interactional, organizational and systemic levels that can influence successful PC and PH collaborations. One of these factors identified at the interactional level was personal values, beliefs, and attitudes. Exploring stakeholders values, beliefs and attitudes about collaboration is of particular interest for this paper. Therefore, the purpose of this study was to explore various stakeholders’ viewpoints about barriers and facilitators of PC and PH collaboration at the systemic, organizational and interactional level).

## Methods

**Figure 1:**
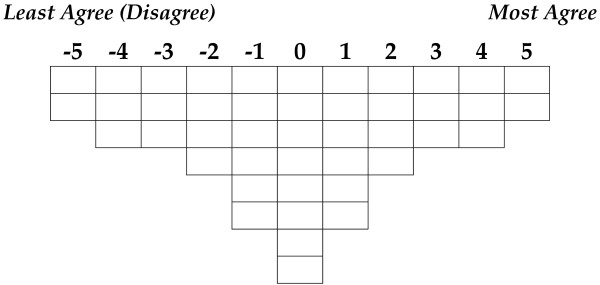
Q-sort table.

We used Q-methodology to identify common viewpoints held by participants who attended an invitational national meeting in Canada to discuss PC and PH collaboration in 2010. This is an exploratory study and was conducted in two phases; in Phase 1 an instrument, a Q-sort table (Figure 1), and a short demographic questionnaire were developed and used in Phase 2 for data collection.

### Q-methodology

Q-methodology was introduced by William Stephenson in 1935 [18,19] and has been employed sporadically since then. The availability of suitable computer programs [20] has widened the use and acceptance of this research method. It is used to identify unique viewpoints, as well as commonly shared views on a research topic and is particularly useful in exploring human perceptions and interpersonal relationships [21]. This methodology has been used in many health-related research areas including the evaluation of job satisfaction of nurses [22], clinical decision making [23], educational program [24], and simulation use in nursing education [25].

Q-methodology has been described as a combination of qualitative and quantitative techniques that allows researchers to identify groups of participants with similar viewpoints [26] where the goal is usually to identify different patterns of thought rather than their numerical distribution among a larger population. As a result, the number or proportion of participants is not the focus of the research; rather the focus is to identify different viewpoints about the topic of study [27].

The current Q-study involved two main phases: Phase 1 included instrument development and Phase II focused on data collection. Each phase is described below.

### Phase 1: instrument development

#### Identification of statements (referred to as the concourse)

As part of the four year program of research, the scoping literature review was completed including papers from Canada, the U.S., the U.K., New Zealand and Australia, from 1988 to 2008 [1,28]. This was followed by interviews with 74 key informants who had experience with or knowledge of PC and PH collaborations. Interviews also focused on identifying barriers and facilitators and the nature of collaborations. The scoping literature review extractions and key informant interviews were coded inductively and organized within three ecological levels (systemic, organizational, interactional). Findings from both studies were summarized into short statements related to factors found to influence collaboration at each level. These statements constructed the concourse for the current Q-methodology study.

The statements in the concourse were reviewed for similarities and differences by the research team that included a Q-methodologist, nursing faculty with experience working with the PH and PC sectors, and a research coordinator. The statements in the concourse were categorized into major themes or domains based on similarities between ideas. Overall, themes were identified at systems, organizational and interactional levels [29]. Major themes at the systems level included: health care structures; government involvement; funding and resources; models of care delivery; professional education and training; power and control; information systems; evidence of benefits of collaboration; and leadership. Organizational level themes addressed: communication; understanding, trust and valuing of the other sector; cross sector planning and implementation processes; organizational team structures; proximity or co-location; funding to support collaboration; organizational leadership; goals, mandates and vision; and information systems and sharing. Interactional level themes were: role clarity; flexibility in roles; trusting relationships; knowledge of each other’s worlds; interpersonal communication processes; approaches to practice; and knowledge and skills of practitioners related to collaboration. Statements within each of these themes were refined by the research team over several meetings. Repetitive responses were deleted and disagreements were resolved through consensus among the research team members. To have a representative sample of the statements (the Q-sample) in the concourse, an inductive process was used in the absence of a pre-existing theoretical hypothesis or framework. Based on the convergence of ideas, statements were merged into one final set of statements (the Q-sample) to be used in Phase 2 of the study. This final Q-sample included 44 statements which represented key ideas from all emerging themes about collaboration between PH and PC.

#### The Q-sort table

After assembling the Q-sample, a grid or Q-sort table was developed with 44 cells equal to the number of statements in the Q-sample (see Figure 1). To pilot test the Q-sort table and statements, one potential participant reviewed the study materials and worked through the Q-sort assigning the statements to the Q-sort table. Minor editing suggestions were adopted to clarify a number of the statements. We recognize that having one individual test the tool is a study limitation. We were unable to recruit additional pilot testers due to tight time limitations between obtaining ethics approval and the national event where we recruited participants.

The final Q-sort table consisted of 44 cells in eight rows and eleven columns of differing lengths (see Figure 1). Anchors of −5 (*least agree* or *disagree*) and +5 (*most agree*) were assigned to the extreme scores of the Q-sort table. The columns between the anchors were numbered sequentially from −5 to +5 with the middle column having a 0-label.

### Phase 2: data collection

In this phase delegates who attended a national meeting focused on PC and PH collaboration were asked to participate in the study and complete the Q-sort. Participants included researchers, policy-makers, directors and managers, and practitioners (e.g., nurses, family physicians, dietitians) from federal agencies and institutions as well as being from the provinces of Nova Scotia, Ontario and British Columbia in Canada. Each consented participant received a package which included detailed instructions and an example of a completed Q-sort. They were not compensated for participating. They were expected to complete the Q-sort by themselves based on the provided instruction. The researchers were also present to answer any questions and clarify the process if needed. The participants were instructed to read the statements and place the number of the statement into the empty cell that corresponded with the degree of agreement the respondent had with each statement. Respondents were not asked to explain their choices due to time limitations since the data collection occurred concurrently with another meeting. Any statement placed under a negative number on the Q-sort table indicated disagreement (or less agreement) and any statement placed under a positive number indicated agreement (or more agreement). The respondents continued in this manner until all blanks on the Q-sort table were filled. The Q-sort was completed by each participant independently.

The Q-sort table was constructed such that only two statements could be placed under −5 and +5, three statements under −4, -3, +3, and +4, four statements under −2 and +2, six statements under −1 and +1, and finally eight statements under the central column 0. Participants were also asked to complete a short demographic survey.

### Sample size (P-set)

In total, 25 individuals including researchers, policy-makers, directors and managers, and practitioners (e.g., nurses, family physicians, dietitians) from federal agencies and institutions as well as from the provinces of Nova Scotia, Ontario and British Columbia in Canada participated in this study. Q-studies typically use small sample sizes compared to quantitative approaches. Low response rates do not bias the results because the primary objective is to identify major viewpoints among participants not their proportion within the larger population [27].

### Data analysis

The analysis of the Q-sorts was conducted with PQMethod 2.33, a free downloadable software program [30]. A by-person factor analysis (i.e., the statistical analysis is performed by person rather than by variable, trait, or statement) of the Q-sorts was conducted to identify groups (factors) of participants with similar viewpoints. Therefore, each group (or factor) represents individuals with similar views, feelings, or experiences about the topic. Each individual with a significant loading (p ≤ .05) on one factor is counted as a member of the group loading on that factor. A factor loading is a correlation between a Q-sort and the factor itself. The standard error (SE) of the correlation is estimated by SE = SQRT(1/N), where N is the number of statements [31]. A correlation is statistically significant if it is ≥ 1.96 times the standard error and all respondents who significantly load on one factor constitute a group of like-minded individuals.

#### Factor extraction and rotation

There are two methods of factor extraction available in the PQMethod 2.33 program: the principal component method and the centroid method. The main difference between these two methods is that in the principal component method the *variance of the loadings* are maximized whereas in the centroid approach the *average of the loadings* are maximized. In addition, two methods of factor rotation are available in this program, varimax and judgmental (or manual) rotation. Usually, rotation methods are informed by the theoretical framework rather than simply using statistical criteria. A manual rotation is used when there is a theoretical framework for latent factors. Since a theoretical framework was not available for this study, the centroid method was used for factor extraction followed by varimax rotation.

Following factor extraction and factor rotation, a weighted (synthetic) Q-sort is produced for each rotated factor by using a weighted averaging method to calculate the score for each statement in the factor array [27]. Then, each factor is typically assigned a name that reflects the factor makeup. Names are assigned to each factor usually based on the factor’s *distinguishing* statements which are statements that score significantly different on that factor compared to any of the other factors. For this study members of the research team met to interpret and name the factors.

### Validity and reliability

The test-retest reliability of the Q-sorting has been found to be 0.80 or higher in some studies [32,33]. Content validity is typically assessed by a team of domain experts. The face validity of the statements is assured by using participants’ own wording of the statements with only slight / minor editing for grammar and readability [26]. For a complete review of Q-methodology, readers are referred to Akhtar-Danesh et al. [26] for practical guidance and to Brown [31] for a theoretical account.

#### Protection of human subjects

Participants were approached to participate in the study after ethical approval was received from the relevant research ethics board.

## Results

Using a by-person factor analysis, three factors (salient viewpoints) emerged, which included 23 individuals. Two participants did not load significantly on any of these three factors and were excluded from further comparative analyses. Factors were named based on their distinguishing statements as follows: a) System Driven Collaborators, b) Cautious Collaborators, and c) Competent Isolationists. There were no statistically significant relationships between these three factors and any of the demographic variables (i.e., sector they represented, level of education, employment status, and years of experience).

### Factors

#### Factor 1: system driven collaborators

Fifteen respondents loaded on this factor; four from Nova Scotia, eight from Ontario, and three from British Columbia. On average they had 24.1 years of experience in the health care profession (SD = 9.8, min = 4, max = 37 years).

This group strongly believed that there is the need for having a clear mandate from the top to enable PH, PC (Statement 34), and the rest of the health system to effectively work together and that people in different branches in the Ministry/Ministries have to really believe in collaboration, support it, and write policies to have organizations work together (Statement 8). Further to this, they more than other groups believed that leadership at the Ministry level was important to “make it happen” (Statement 19) In addition, they strongly supported the statement that “I think we need models like community health centres which are globally funded (salaried physicians who work in a team setting with a range of health professionals – nurses, nutritionists, social workers) (Statement 38). So the more we move into this kind of model, PC and PH collaborations might become richer” (Statement 38). On the other hand, they strongly disagreed that “differing mandates are a barrier to collaboration. PH can’t provide individual care because they are population health-based and group-based. For example PH is working on healthy food policies and trying to work with schools”(Statement 25). This group also did not feel that unions (Statement 24) or funding differences between PC and PH (Statement 44) are a particular challenge to collaboration. Generally, statements that were perceived in the neutral range by this group as compared to the other two groups were statements that reflected organizational (Statements 1, 23, 32) and interactional (Statement 4) level themes. This further supports the perspective of this group being ‘system driven collaborators’. They perceived system level themes as most influential for PC and PH collaboration (See Table 1 for statements which distinguish this factor from the others).

**Table 1:** Distinguishing statements for factor 1

**No.**	**Statement**	**Theme**	**Factor 1**	**Factor 2**	**Factor 3**
34	We need to have a clear mandate from the top to enable public health, primary care and the rest of the health system to work together more effectively.	Systemic	**5**	−4	0
8	I think that people in different branches in the Ministry/ Ministries have to really believe in collaboration and support it enough so that they write policies that say these organizations are going to work together.	Systemic	**4**	0	−2
38	I think we need models like community health centres which are globally funded (salaried physicians who work in a team setting with a range of health professionals – nurses, nutritionists, social workers). So the more we move into this kind of model, primary care and public health collaborations might become richer.	Systemic	**4**	−1	1
33	I think the base unit of the health care system, just as WHO and everybody else around the world suggests, should be some sort of community health centre model which provides a primary care range of services practicing in the context of community.	Systemic	**3**	−2	0
35	I think without knowing what one another does and how we can actually utilize one another, we are really actually providing a disservice to communities that we serve.	Interactional	**2**	4	5
19	I think you need to have someone in the Ministry who believes a collaborative structure is important and would make it happen.	Systemic	**2**	−1	0
32	It’s a problem when there is a lack of involvement of all parties in the planning stages. For example, when middle management is not involved in the decision making process or we need the people who are going to be delivering the programming when it hits the ground at the table.	Organizational	**1**	3	4
4	Partners need to consistently engage in dialogue to resolve issues. For example, they are working together identifying specific patients that both are involved with.	Interactional	**1**	3	−1
1	I think we need to move more toward use of the electronic health record where all collaboration partners have full access to every chart. Then everyday communication around patient care would be much better.	Organizational	**0**	−2	−2
23	I think an important facilitator of collaboration is having a memorandum of understanding (MOU) of how we work together. For example, MOU says that each partner agrees to put X hours of service in on a weekly basis and we will have a planning day every year.	Organizational	**−1**	2	−3
44	I think a problem in collaborations is that there are funding differences between primary care and public health systems, namely, primary care has a lot more money and people than public health. That’s a built-in challenge to any kind of collaboration.	Systemic	**−2**	2	4
24	Public health is largely in a unionized environment and is a bigger, institutional culture. They’ve got much more prescribed practices around how they can deploy staff which is a big barrier to collaboration.	Organizational	**−3**	−2	0
25	I think differing mandates are a barrier to collaboration. Public health can’t provide individual care because they are population health-based and group-based. For example public health is working on healthy food policies and trying to work with schools.	Systemic	**−4**	−2	3

#### Factor 2: cautious collaborators

Five individuals, all from the province of Ontario, loaded on the second factor. They strongly agreed that, “we need to have a better consciousness-raising about what collaborations might be possible and would be beneficial, and also reflect on the collaborations that we already have” (Statement 36). They also highly agreed with the statements that: “There are turf protection issues. PH wants to make sure that they don’t get swallowed up by PC issues. They want to deal with issues at a population level as opposed to an individual health level” (Statement 41). Another subject they strongly agreed with was that “everybody feels that they are at capacity and there’s no room for anything more such as working on a collaboration” (Statement 14). They strongly disagreed that having a clear mandate from the top would enable PH and PC and health system collaboration (Statement 34). They also opposed the idea that “for better communication there has to be availability of electronic communication mechanisms between PC and PH sectors (e.g. email listservs to share information about free mental health sessions in the community)” (Statement 15). Related to this, they disagreed that there is a lack of communication between government agencies, since integration of high tech communications are still in its infancy (Statement 42). But at the local level, this group felt that “partners need to consistently engage in dialogue to resolve issues”, such as the patients they are both involved in, for successful collaboration (Statement 4). In general, this group felt strategies for successful collaboration were focused at the local level, such as consistent dialogue with partners (Statement 4), starting small (Statement 43), and having a memorandum of understanding (Statement 23) (See Table 2 for statements which distinguish this group from the others).

**Table 2:** Distinguishing statements for factor 2

**No.**	**Statement**		**Factor 1**	**Factor 2**	**Factor 3**
36	We need to have a better consciousness-raising about what collaborations might be possible and would be beneficial, and also reflect on the collaborations that we already have.	Organizational	2	**5**	2
41	There are turf protection issues. Public health wants to make sure that they don’t get swallowed up by primary care issues. They want to deal with issues at a population level as opposed to an individual health level.	Systemic/Organizational	0	**4**	1
14	Everybody feels that they are at capacity and there’s no room for anything more such as working on a collaboration.	Organizational	−2	**4**	−3
4	Partners need to consistently engage in dialogue to resolve issues. For example, they are working together identifying specific patients that both are involved with.	Interactional	1	**3**	−1
43	What fosters collaboration at the organizational level is if we can keep it small to start.	Organizational	−1	**2**	0
23	I think an important facilitator of collaboration is having a memorandum of understanding (MOU) of how we work together. For example, MOU says that each partner agrees to put X hours of service in on a weekly basis and we will have a planning day every year.	Organizational	−1	**2**	−3
8	I think that people in different branches in the Ministry/ Ministries have to really believe in collaboration and support it enough so that they write policies that say these organizations are going to work together.	Systemic	4	**0**	−2
22	I think the fee-for-service model doesn’t work. We need to have money attached in a way that fosters collaboration. To really get doctors to pay attention beyond their practice and their individual patients, we have to pay them differently if we want them to do different work.	Systemic	4	**0**	3
31	I think the fee-for-service physician model is a disincentive to collaboration. For example, it is a disincentive to meet with collaborators during billable office hours.	Systemic	3	**0**	2
38	I think we need models like community health centres which are globally funded (salaried physicians who work in a team setting with a range of health professionals – nurses, nutritionists, social workers). So the more we move into this kind of model, primary care and public health collaborations might become richer.	Systemic	4	**−1**	1
24	Public health is largely in a unionized environment and is a bigger, institutional culture. They’ve got much more prescribed practices around how they can deploy staff which is a big barrier to collaboration.	Organizational	−3	**−2**	0
25	I think differing mandates are a barrier to collaboration. Public health can’t provide individual care because they are population health-based and group-based. For example public health is working on healthy food policies and trying to work with schools.	Systemic	−4	**−2**	3
33	I think the base unit of the health care system, just as WHO and everybody else around the world suggests, should be some sort of community health centre model which provides a primary care range of services practicing in the context of community.	Systemic	3	**−2**	0
42	The lack of communication between the various government agencies is obvious just from the large number of faxes that come through. So integration of high tech communication is in its infancy and needs to be improved.	Systemic	−1	**−3**	−1
21	I think it is easy to get people in all branches/departments of the Ministry/Ministries to recognize the importance of public health and prevention.	Systemic	−5	**−3**	−5
34	We need to have a clear mandate from the top to enable public health, primary care and the rest of the health system to work together more effectively.	Systemic	5	**−4**	0
15	For better communication there has to be availability of electronic communication mechanisms between public health and primary care. ( e.g. email listservs to share information about free mental health sessions in the community).	Organizational	0	**−4**	0

#### Factor 3: competent isolationists

Only three individuals loaded significantly on this factor (one from each province). They strongly believed that it is necessary for PC and PH sectors to spend time to make sure that both parties clearly understand the differences between their roles (Statement 13). They believed that physicians, nurses and social workers will not see the value in collaboration because they don’t share courses during their professional education (Statement 40). They also agreed that it is important for people to use the skill sets that they have rather than learn new ones, and to use skills sets others have in collaborations (Statement 7). They also believed different mandates are barriers to collaboration and that PH cannot provide individual care, because they are population health-based and group-based (Statement 25). Also they believed that, “collaboration won’t work if people haven’t got the stable and sustainable funding to get it established, evaluated and carry it on” (Statement 6). They also strongly disagreed that there is evidence on the benefits of collaboration related to long term health benefits for individuals in the population (Statement 17). They also did not see value in having memorandums of understanding in collaborations (Statement 23), people in Ministry branches writing policies instructing PC and PH to work together (Statement 8), or a PH staff presence in PC settings (Statement 30). They did not particularly feel that being physically co-located (Statement 2) or consistently engaged in dialogue (Statement 4) would be helpful. In other words, respondents in this group did not seem convinced about the value of collaboration, identified many barriers at all levels as well as differences between PC and PH (See Table 3).

**Table 3:** Distinguishing statements for factor 3

**No.**	**Statement**		**Factor 1**	**Factor 2**	**Factor 3**
13	We need to spend time making sure that both parties clearly understand the difference between the roles of primary care organization and the roles of the Public Health organization.	Interactional	0	1	**5**
40	Physicians, nurses and social workers are not sharing courses when they’re being educated; so they are not going to see the value of working collaboratively.	Systemic	−1	0	**4**
25	I think differing mandates are a barrier to collaboration. Public health can’t provide individual care because they are population health-based and group-based. For example public health is working on healthy food policies and trying to work with schools.	Organizational	−4	−2	**3**
6	Collaboration won’t work if people haven’t got the stable and sustainable funding to get it established, evaluated and carry it on.	Organizational	0	1	**3**
7	I think it is important in a collaboration that people use the skill set that they have. They do not always have to learn new skill sets, but utilize the skill sets that other people have.	Interactional	−1	−1	**2**
38	I think we need models like community health centres which are globally funded (salaried physicians who work in a team setting with a range of health professionals – nurses, nutritionists, social workers). So the more we move into this kind of model, primary care and public health collaborations might become richer.	Systemic	4	−1	**1**
3	It’s a lot about relationships and trust. People need to trust one another and know that everybody is working towards the same end. That will have the biggest impact on collaboration.	Interactional	5	5	**1**
34	We need to have a clear mandate from the top to enable public health, primary care and the rest of the health system to work together more effectively.	Systemic	5	−4	**0**
24	Public health is largely in a unionized environment and is a bigger, institutional culture. They’ve got much more prescribed practices around how they can deploy staff which is a big barrier to collaboration.	Organizational	−3	−2	**0**
33	I think the base unit of the health care system, just as WHO and everybody else around the world suggests, should be some sort of community health centre model which provides a primary care range of services practicing in the context of community.	Systemic	3	−2	**0**
2	I think physical co-location of primary care and public health results in increased exposure to one another and therefore a stronger understanding of each other’s skills and roles.	Organizational	3	1	**−1**
4	Partners need to consistently engage in dialogue to resolve issues. For example, they are working together identifying specific patients that both are involved with.	Interactional	1	3	**−1**
30	A facilitator for collaboration would be having a public health staff presence in a primary care setting—so there’s a face to public health. I can get information without having to go through a complicated process.	Organizational	1	1	**−2**
8	I think that people in different branches in the Ministry/ Ministries have to really believe in collaboration and support it enough so that they write policies that say these organizations are going to work together.	Systemic	4	0	**−2**
23	I think an important facilitator of collaboration is having a memorandum of understanding (MOU) of how we work together. For example, MOU says that each partner agrees to put X hours of service in on a weekly basis and we will have a planning day every year.	Organizational	−1	2	**−3**
17	We have evidence on the benefits of collaboration that are linked to long term health benefits for individuals in the population.	Systemic	−2	0	**−4**

### Consensus statements

There were several statements with which all participants equally agreed or disagreed (Table 4). For example, they all believed that lack of vision in collaboration is a barrier and that people are not clear on the end results of collaboration (Statement 10). They all strongly disagreed that “politicians have research evidence to say that collaboration will save money so will put money behind it” (Statement 11) and that “mutual respect between PC and PH sectors is not necessarily required for effective collaboration” (Statement 39). Patient confidentiality was not seen as an area of concern (Statement 37) but respondents felt that different work processes (pace, stress level), such as clinical services work are a barrier (Statement 5). Finally, they all disagreed that: “In a provincial healthcare system, you have to have the primary care and public health players in the collaboration working for the same entity-- for the same overall administrative structure (Statement 29).

**Table 4:** Consensus statements

**No.**	**Statement**	**Factor 1**	**Factor 2**	**Factor 3**
5	I think different work processes are a barrier to collaboration. For example, staff who work in clinical services work at the very usually stressful sort of primary care pace. Colleagues who work in other areas of Public Health aren’t in the same mindset, stress level, pace level.	−4	−5	−2
10	A lack of vision in collaborations is a barrier. For example, people are not being clear on what the end result is going to be.	2	3	2
11	I think politicians have research evidence to say that collaboration will save money so will put money behind it.	−4	−4	−4
16	There’s a strong lack of collaboration for prevention interventions. Primary care and public health work in silos. I think we need to break those silos.	1	1	1
18	If primary care and public health professionals are so married to how they interpret their role and mandate, that a person can’t step outside of that role if the situation calls for it, it can be a barrier to collaboration. People need to be comfortable with a blurring of the lines.	1	2	0
28	In collaborations there is a threat that public health staff who don’t have a primary care background are moving into situations where they’re going to have to deal with primary care issues.	−2	−1	−1
29	In a provincial healthcare system, you have to have the primary care and public health players in the collaboration working for the same entity-- for the same overall administrative structure.	−3	−3	−4
37	I think the issue of patient confidentiality and privacy is a huge area of concern when working in a collaboration.	−3	−1	−3
39	Mutual respect between primary care and public health sectors is not necessarily required for effective collaboration.	−5	−5	−5

In addition to the statements presented in Tables 1, 2, 3, 4, a list of all statements used in this study is presented in Table 5.

**Table 5:** List of statements in the Q-sample

**Statement number**	**Statement**
1	I think we need to move more toward use of the electronic health record where all collaboration partners have full access to every chart. Then everyday communication around patient care would be much better.
2	I think physical co-location of primary care and public health results in increased exposure to one another and therefore a stronger understanding of each other’s skills and roles.
3	It’s a lot about relationships and trust. People need to trust one another and know that everybody is working towards the same end. That will have the biggest impact on collaboration.
4	Partners need to consistently engage in dialogue to resolve issues. For example, they are working together identifying specific patients that both are involved with.
5	I think different work processes are a barrier to collaboration. For example, staff who work in clinical services work at the very usually stressful sort of primary care pace. Colleagues who work in other areas of Public Health aren’t in the same mindset, stress level, pace level.
6	Collaboration won’t work if people haven’t got the stable and sustainable funding to get it established, evaluated and carry it on.
7	I think it is important in a collaboration that people use the skill set that they have. They do not always have to learn new skill sets, but utilize the skill sets that other people have.
8	I think that different branches in the Ministry/ Ministries have to really believe in collaboration and support it enough so that they write policies that say these organizations are going to work together.
9	For better collaboration we need to define roles, where everyone fits in the big picture. It is not about turf and it’s not about ‘I can do this better than you’. It’s about how can we deliver cost effective care to the patient the best way.
10	A lack of vision in collaborations is a barrier. For example, people are not being clear on what the end result is going to be.
11	I think politicians have research evidence to say that collaboration will save money so will put money behind it.
12	I don’t see any formal linkage between the public health nurses and the primary care physicians and there is no support at the higher systems level for that to happen.
13	We need to spend time making sure that both parties clearly understand the difference between the role of primary care organization and the role of the Public Health organization.
14	Everybody feels that they are at capacity and there’s no room for anything more such as working on a collaboration.
15	For better communication there has to be availability of electronic communication mechanisms (i.e. email listserv) between public health and primary care. (i.e., for information sharing about free mental health sessions in the community).
16	There’s a strong lack of collaboration for prevention interventions. Primary care and public health work in silos. I think we need to break those silos.
17	We have evidence on the benefits of collaboration that are linked to long term health benefits for individuals in the population.
18	If primary care and public health professionals are so married to how they interpret their role and mandate, that a person can’t step outside of that role if the situation calls for it, it can be a barrier to collaboration. People need to be comfortable with a blurring of the lines.
18	In collaborations there is a threat that public health staff who don’t have a primary care background are moving into situations where they’re going to have to deal with primary care issues.
19	I think you need to have someone in the Ministry that believes a collaborative structure is important and would make it happen.
20	There is limited evidence of effectiveness of collaboration. I think evaluations should occur regularly and collaborators should keep talking all the way along.
21	I think it is easy to get people in all branches/departments of the Ministry/Ministries to recognize the importance of public health and prevention.
22	I think the fee-for-service model doesn’t work. We need to have money attached in a way that fosters collaboration. To really get doctors to pay attention beyond their practice and their individual patients, we have to pay them differently if we want them to do different work.
23	I think an important facilitator of collaboration is having a memorandum of understanding (MOU) of how we work together. For example, MOU says that each partner agrees to put X hours of service in on a weekly basis and we will have a planning day every year.
24	Public health is largely in a unionized environment and is a bigger, institutional culture. They’ve got much more prescribed practices around how they can deploy staff which is a big barrier to collaboration.
25	I think differing mandates are a barrier to collaboration. Public health can’t provide individual care because they are population health-based and group-based., For example public health is working on healthy food policies and trying to work with schools.
26	I think collaboration needs contributions in-kind from each party of their own staff and resources as well as additional resources.
27	Public Health is organized by programs and not geography necessarily. We need to align more geographically so we can start working a little more closely with our primary care and community partners.
29	In a provincial healthcare system, you have to have the primary care and public health players in the collaboration working for the same entity-- for the same overall administrative structure.
30	A facilitator for collaboration would be having a public health staff presence in a primary care setting—so there’s a face to public health. I can get information without having to go through a complicated process.
31	I think the fee-for-service physician model is a disincentive to collaboration. For example, it is a disincentive to meet with collaborators during billable office hours.
32	It’s a problem when there is a lack of involvement of all parties in the planning stages. For example, when middle management is not involved in the decision making process or we need the people who are going to be delivering the programming when it hits the ground at the table.
33	I think the base unit of the health care system, just as WHO and everybody else around the world suggests, should be some sort of community health centre model which provides a primary care range of services practicing in the context of community.
34	We need to have a clear mandate from the top to enable public health, primary care and the rest of the health system to work together more effectively.
35	I think without knowing what one another does and how we can actually utilize one another, we are really actually providing a disservice to communities that we serve.
36	We need to have a better consciousness-raising about what collaborations might be possible and would be beneficial, and also reflect on the collaborations that we already have.
37	I think the issue of patient confidentiality and privacy is a huge area of concern when working in a collaboration.
38	I think we need models like community health centres which are globally funded (salaried physicians who work in a team setting with a range of health professionals – nurses, nutritionists, social workers). So the more we move into this kind of model, primary care and public health collaborations might become richer.
39	Mutual respect between primary care and public health sectors is not necessarily required for effective collaboration.
40	Physicians, nurses and social workers are not sharing courses when they’re being educated; so they are not going to see the value of working collaboratively.
41	There are turf protection issues. Public health wants to make sure that they don’t get swallowed up by primary care issues. They want to deal with issues at a population level as opposed to an individual health level.
42	The lack of communication between the various government agencies is obvious just from the large number of faxes that come through. So integration of high tech communication is in its infancy and needs to be improved.
43	What fosters collaboration at the organizational level is if we can keep it small to start.
44	I think a problem in collaborations is that there are funding differences between primary care and public health systems— namely, primary care has a lot more money and people than public health. That’s a built-in challenge o any kind of collaboration.

## Discussion

Based on consensus statements, PC and PH policy makers, managers, practitioners and researchers held a common view that a lack of vision for collaboration, where people are not clear on the end result of collaboration, can be a significant barrier to collaboration. This has been corroborated by others [10,34,35]. Therefore, it is imperative that the vision for any collaboration be determined early on and clearly communicated across all levels and among partners in collaborations, ranging from executive directors to front line staff.

Our results also showed that participants all strongly disagreed that “politicians have research evidence to say that collaboration will save money so will put money behind it”. This implies that researchers must work with policy makers to ensure that evidence related to the outcomes of collaborations are disseminated effectively to both sectors, including provincial/state and national health leaders who have the power to make such policy decisions. Think tanks were recently held in three Canadian provinces involving researchers, policy makers, managers and practitioners to share collective results and determine future action for practice, policy and research based on our program of research on PC and PH collaboration. However, it is too soon to say how these events have impacted action towards collaboration. Researchers are encouraged to use integrated knowledge exchange approaches [36] in future research, which demands active involvement of decision-makers on research teams to support research directions that are relevant to practice and policy and to increase uptake of results.

Perspectives of participants evaluated in this study correspond to three distinct groups, each representing an important and differing viewpoint regarding collaboration between PH and PC sectors in Canada. Each of the three groups was given a descriptive title based on their distinguishing statements, i.e., *System Driven Collaborators*, *Cautious Collaborators*, and *Competent Isolationists*.

*System Driven Collaborators* held common views that system level influences, such as provincial level policies mandating PC and PH collaboration, and globally funded (non fee-for- service) PC physician payment models can have a significant positive impact on PC and PH collaboration. Therefore, policy makers need to develop policies mandating PC and PH collaboration and encourage expansion of salaried physician payment models moving away from fee-for-service payment structures. Similar views related to macro system changes are supported by federal U.S. agencies which have shown strong leadership towards PC and PH integration including the Centre for Disease Control (CDC) -the main public health agency in the federal government-- as well as the Health Resources and Services Agency (HRSA)- the largest PC service agency responsible for PC and PH workforce development and PC services delivered through community health centers [37]. A recent Institute of Medicine (IOM) recommendation directs the secretary of HHS (Health Human Services) to:

“work with all agencies within the department as a first step in the development of a national strategy and investment plan for the creation of PC and PH infrastructure strong enough and appropriately integrated to enable the agencies to play their appropriate roles in furthering the nations’ population health goals.” (p149) [2].

*Cautious Collaborators,* who were all from the province of Ontario, strongly agreed that although we need to gain a better awareness of what collaborations might be possible and beneficial, we need to be cautious about moving forward on them. This group is concerned about the threat of a reduction of public health’s workforce which is already over-stretched for working on population health approaches. The fear is that PH will be swallowed up by the PC sector, which is primarily focused on individual health. This group did not feel that provincial mandates to collaborate would be helpful. These results are not very surprising given that Ontario is the only Canadian province in which 36 health units exist independent of a regional health authority [38]. Given these results, managers in PH and PC are advised to collaboratively develop work plans where a mix of population and individual approaches might work synergistically to address local community needs. For example, PH could apply their expertise in prevention to increase PC capacity related to chronic disease prevention, such as obesity prevention [39,40], which has been shown to be a promising area for PC and PH collaboration.

A small group of *Competent Isolationists* held strong beliefs that PC and PH sectors need to clearly understand the differences between each other’s roles, which were viewed as being separate and distinct (population and group based versus individually based). This group also believed that collaboration would not work without stable, sustainable funding and that multi-disciplinary professionals would not see value in collaboration, since they do not share educational programs. This latter finding may be explained by a recent historical review by Scutchfield and colleagues [41] of PC and PH collaboration. They describe the entrenched professional culture of PC physicians as autonomous professionals with strong personal accountability to their patients as barriers to shifting to inter- and intra-organizational collaborative teams in practice. Given this set of views about collaboration, managers and policy makers should be aware that some stakeholders, the *Competent Isolationists*, will be skeptical about collaboration and will likely only buy-in if evidence of collaboration effectiveness is demonstrated and sustainable funding is provided. These views also inform educators of the need to ensure that interprofessional education goes beyond gaining increased understanding of roles of various disciplines, but also needs to include enhanced understanding of professionals’ roles in PC and PH sectors. These sectors are often ignored and students in the health professions have minimal or no exposure to them in their curricula. Perhaps positive gains can be made in interprofessional education with greater exposure to inter-organizational collaboration.

Further, *Cautious Collaborators* did not believe that there was evidence of long term health benefits to be gained from collaboration. Varda, Shoup and Miller [42] conducted a systematic review exploring the public affairs literature related to collaborations. Based on their results, they concluded that PH leaders need to be able to analyse outcomes of their collaborations to determine if the costs of developing them are worth the resources spent. Also noted by Landon et al., rigorous and systematic assessments of integration are essential to assess the impact of PC and PH collaboration on the health of communities [37]. Therefore, collaboration partners are strongly encouraged, at the very least, to co-define process and outcome expectations in advance and incorporate evaluations into their partnership work. This would be particularly relevant and important for the *Competent Isolationists*.

Our study had some limitations. The Q-sort was pilot tested by only one individual, which may have resulted in statements or instructions being unclear. However, during data collection, the research team was available to answer any questions and clarify the meaning of statements. There were very few questions raised during data collection. Further, the Q-sort was conducted at the end of a long day at a national meeting related to PC and PH collaboration. Participants may have had limited energy to critically think through the statements. However, participants had just learned about Q-methodology as an innovative research method before the data collection took place, and most showed great interest in the approach. This may have resulted in greater attention to the task of sorting statements. We also did not have an opportunity to check with participants if they agreed with our interpretation of results and naming of the factors.

## Conclusions

Viewpoints held by key stakeholders have the potential to influence a collaboration’s success both positively and negatively. Understanding differences in views is critical to managing change processes both in initiating and sustaining collaborations. Whether stakeholders are working at systemic (federal, provincial/state policy makers), organizational (regional or local managers) or interactional (front line practitioners) levels, their viewpoints must be considered and addressed to successfully move collaborations forward. A mix of *Competent Isolationists, Cautious Collaborators and System-driven Collaborators* are likely found in most jurisdictions and at all levels; therefore, multiple approaches to support collaboration will be required to address partners’ varying concerns. This paper provides a window into seeing and understanding common viewpoints held by stakeholders in Canada. One caution should be considered: Since *Cautious Collaborators* were all from a province that organizes PH into independent organizations, readers are encouraged to consider the potential impact of structural context of PC and PH systems in their own jurisdictions and assess how it might impact the viewpoints of its collaborators. As noted in the recent IOM report on primary care and public health integration, aligned leadership with capacity to initiate and manage change is an essential principle for successful PC PH integration [2]. As with any effective change management strategy, having a good understanding of views of health care leaders at all levels is essential for success.

## Abbreviations

PC: Primary care; PH: Public health.

## Competing interests

The authors have no competing interests related to this work.

## Authors’ contributions

NAD contributed in the conceptualization and design of the study, development of the Q-sort tool, analysis of Q-sorts, and preparation of the manuscript. RV contributed in the conceptualization and design of the study, development of the Q-sort tool, obtaining ethics approval, recruitment, and data interpretation as well as the preparation and review of this manuscript. LO contributed in the conceptualization and design of the study, development of the Q-sort tool, data interpretation as well as the preparation and review of this manuscript. PA contributed in the conceptualization and design of the study, development of the Q-sort tool, data interpretation as well as the preparation and review of this manuscript. VM contributed in the preparation and review of this manuscript. All authors read and approved the final manuscript.

## Pre-publication history

The pre-publication history for this paper can be accessed here:

http://www.biomedcentral.com/1472-6963/13/311/prepub
